# Dynamics of Circulating γδ T Cell Activity in an Immunocompetent Mouse Model of High-Grade Glioma

**DOI:** 10.1371/journal.pone.0122387

**Published:** 2015-05-08

**Authors:** Benjamin H. Beck, Hyunggoon Kim, Rebecca O’Brien, Martin R. Jadus, G. Yancey Gillespie, Gretchen A. Cloud, Neil T. Hoa, Catherine P. Langford, Richard D. Lopez, Lualhati E. Harkins, Lawrence S. Lamb Jr.

**Affiliations:** 1 Department of Medicine, University of Alabama at Birmingham School of Medicine, Birmingham, Alabama, 35294, United States of America; 2 Department of Pathology, University of Alabama at Birmingham School of Medicine, Birmingham, Alabama, 35294, United States of America; 3 Integrated Department of Immunology, National Jewish Health, Denver, Colorado, United States of America; 4 Department of Pathology, University of California Irvine/Veterans Affairs Medical Center/Long Beach, Long Beach, California, 80206, United States of America; 5 Department of Neurosurgery, University of Alabama at Birmingham School of Medicine, Birmingham, Alabama, 35294, United States of America; 6 UAB Comprehensive Cancer Center, University of Alabama at Birmingham School of Medicine; Birmingham, Alabama, 35294, United States of America; University Hospital of Navarra, SPAIN

## Abstract

Human γδ T cells are potent effectors against glioma cell lines *in vitro* and in human/mouse xenograft models of glioblastoma, however, this effect has not been investigated in an immunocompetent mouse model. In this report, we established GL261 intracranial gliomas in syngeneic WT C57BL/6 mice and measured circulating γδ T cell count, phenotype, Vγ/Vδ repertoire, tumor histopathology, NKG2D ligands expression, and T cell invasion at day 10–12 post-injection and at end stage. Circulating γδ T cells transiently increased and upregulated Annexin V expression at post-tumor day 10–12 followed by a dramatic decline in γδ T cell count at end stage. T cell receptor repertoire showed no changes in Vγ1, Vγ4, Vγ7 or Vδ1 subsets from controls at post-tumor day 10–12 or at end stage except for an end-stage increase in the Vδ4 population. Approximately 12% of γδ T cells produced IFN-γ. IL-17 and IL-4 producing γδ T cells were not detected. Tumor progression was the same in TCRδ^-/-^ C57BL/6 mice as that observed in WT mice, suggesting that γδ T cells exerted neither a regulatory nor a sustainable cytotoxic effect on the tumor. WT mice that received an intracranial injection of γδ T cells 15m following tumor placement showed evidence of local tumor growth inhibition but this was insufficient to confer a survival advantage over untreated controls. Taken together, our findings suggest that an early nonspecific proliferation of γδ T cells followed by their depletion occurs in mice implanted with syngeneic GL261 gliomas. The mechanism by which γδ T cell expansion occurs remains a subject for further investigation of the mechanisms responsible for this immune response in the setting of high-grade glioma.

## Introduction

T cells expressing γ and δ T cell receptor (TCR) chains represent a small subset (2%–10%) of circulating T cells and, in contrast to αβ T cells, recognize antigens directly without any requirement for antigen processing and presentation on major histocompatibility complex (MHC) molecules [1,2]. Previous studies over the past two decades point to a broad role for γδ T cells in tumor immunosurveillance. Genetically-engineered γδ T cell—deficient mice are highly susceptible to induction of cutaneous carcinogenesis [3]. Similarly, prostate cancer growth is accelerated in γδ T cell-deficient TRAMP mice when compared with fully immunocompetent TRAMP mice [4]. Tumor-infiltrating γδ T cells have been documented in a variety of malignancies including lung cancer [5], renal cell carcinoma [6], seminoma [7], and breast cancer [8] and will recognize and kill tumor cells such as Daudi Burkitt’s lymphoma [9,10], glioblastoma [11,12], neuroblastoma [13], and lung cancer [14,15]. Homeostatic reconstitution of supra-normal numbers of γδ T cells protects against relapse in allogeneic bone marrow transplant patients[16–18].

In both mice and humans, γδ T cells recognize stress-induced antigens via the TCR and/or the activating receptor NKG2D [19]. Ligands for the NKG2D receptor (NKG2DL) include MHC class I-related chain A or B (MICA or MICB) and the UL-16 binding proteins (ULBP1–6) in humans and H60, MULT-1, and RAE-1 in mice. Malignant high-grade gliomas in both mice and humans express several NKG2DL [20,21] and would appear to be targets for γδ T cell attack. Indeed, our previous work has revealed that γδ T cells exhibit strong cytotoxic activity against several GBM cell lines and primary explant cultures[22,23]. Normal astrocytes do not express NKG2DL and therefore are not affected [11,12,24]. When injected into athymic nude mice implanted with human GBM xenografts, *ex vivo* expanded/activated human γδ T cells slowed progression and extended survival [25].

The functional properties of γδ T cells have not been investigated in a fully immunocompetent mouse model of high-grade glioma. Although our findings to date have shown γδ T cells to be cytotoxic effectors against GBM, the known pleiotropic properties of γδ T cells could result in the acquisition of regulatory as well as effector potential, opening the possibility that γδ T cells may also suppress immune responses [26,27]. Indeed, Peng [28] described potent immunosuppression derived from a subset of tumor-infiltrating Vδ1+ T cells from breast and prostate tumors. In this study, we present evidence for a transitory γδ T cell-mediated immune response occurring shortly after tumor engraftment in asymptomatic mice followed by a decline over the course of tumor progression. We also draw parallels to human GBM to describe the dynamic interplay between γδ T cells and high-grade gliomas.

## Materials and Methods

### Mice

C57BL/6 wild-type mice, C57BL/6 TCRδ-deficient (TCRδ^-/-^) mice (B6.129P2-TCRδ^tm1Mom/J^ mice, and C57BL/6 TCRβ-deficient (B6.129P2TCRβ^tm1Mom/J^) mice were all purchased from The Jackson Laboratory. All mice were maintained in pathogen-free facilities in the Brain Tumor Animal Models (BTAM) Facility. This study was carried out in strict accordance with the recommendations in the Guide for the Care and Use of Laboratory Animals of the National Institutes of Health. The protocol was specifically approved by the Animal Care and Use Committee at the University of Alabama at Birmingham (Birmingham, AL). (APN130908793). All surgery was performed under ketamine/xylazine anesthesia, and all efforts were made to minimize suffering.

### Intracranial tumors

Intracranial gliomas were generated using 5 x 10^5^ GL261 murine glioma tumor cells suspended in 5% methylcellulose in serum-free medium. The cells were drawn into a 250μl Hamilton gas-tight syringe mounted in a Chaney repeating dispenser and fitted with a 30G ½-inch needle with a calibrated depth of 2.5 mm from the middle of the bevel opening. Under an operating microscope, the fascia on the skull of the anesthetized mouse were scraped off and a 0.5mm burr hole made 2mm to the right of the midline suture and 1mm caudal to the coronal suture. The syringe was inserted into a Kopf stereotactic electrode clamp mounting bracket attached to an electrode manipulator (David Kopf Instruments; Tujinga, CA) mounted on a Kopf stereotactic frame electrode A-P zeroing bar (#1450). Each mouse was positioned on the stereotactic frame and the needle inserted to the depth marker into the right cerebral hemisphere. Approximately 90–120 seconds after injection of 5μl, the needle was slowly withdrawn over the next minute. The burr hole was plugged with sterile bone wax and skin is closed with Tissu-Mend surgical adhesive (Stryker Orthopedics; Kalamazoo, MI). Tumor engraftment was confirmed by assessment of luminescence. Mice received one intraperitoneal injection of 25mg/kg D-luciferin (Xenogen Corp., Alameda, CA, USA) in PBS. After 10 min the mice were anesthetized and placed in a light-tight box under the cryogenically cooled IVIS camera (Xenogen Corp.). Bioluminescence images are recorded between 10 and 20 min post luciferin administration. The bioluminescence intensity is quantified with the Living Image software (Xenogen) and signal intensity is quantified as the sum of detected photons per second within the region of interest using the Living Image software package. The major endpoint in this study was animal survival; moribund animals that became unresponsive to mild external stimuli were euthanized and this date was used as an estimate of the date of death.

### Microscopy

Formalin-fixed paraffin-embedded (FFPE) sections of GL261 tumors were sectioned and stained with hematoxylin and eosin. For immunohistochemical staining for T cell infiltration, deparaffinized sections were post fixed in 4% neutral buffered formalin followed by antigen retrieval with Citra Plus (Biogenex Laboratories, Freemont CA). Sections were blocked sequentially with avidin, biotin (Biogenex Laboratories, Freemont CA) and FC receptor blocker (Innovex Biosciences, Richmond CA) for 20 minutes at RT. Primary antibody (anti-CD3) was applied at 5μg/ml overnight at 4°C. Multilink secondary antibody (Biogenex Laboratories, Freemont CA) was applied for 30 minutes at RT, followed by Streptavidin-labeled peroxidase (Biogenex laboratories, Fremont CA) for 30 minutes. The immunostaining was developed with Turbo DAB chromogen (Innovex Biosciences, Richmond CA) for 2 minutes or until signal appeared.

For transmission electron microscopy (TEM), tissue samples were fixed in 1/2 Karnovsky's fixative (2% glutaraldehyde and 2.5% paraformaldehyde in 0.1M cacodylate buffer). Samples were post-fixed in 1% Osmium tetroxide, dehydrated in acetone, and embedded in Epon 812 resin. Ultrathin sections were cut using a Leica EM-UC-7 and stained with uranyl acetate and Reynolds’s lead citrate. Images were taken using a Hamamatsu digital camera on a FEI Tecnai T-12 electron microscope. For confocal microscopy, GL261 cells were incubated in media overnight (16–18 hrs) on sterile cover glasses within 24 well plates. The cells were fixed with 2% paraformaldehyde. The cells were then stained on coverslips to detect extracellular proteins with wheat germ agglutinin. Finally, the cells were washed three times in PBS and mounted with ProLong Gold antifade reagent (Invitrogen, Carlsbad, CA). Samples were imaged using a Nikon two laser (HeNe and Argon) PCM 2000 Confocal System on an Eclipse E800 Microscope. Fluorescent dyes in the labeled sample were simultaneously acquired through a single illumination and detection pinhole using Compix Simple PCI software as previously reported (33, 47).

### Culture and activation of γδ T cells

Expansion and activation cultures for cytotoxicity assays and immunotherapy studies were initiated using spleen cells obtained from C57BL/6 TCRβ-deficient (TCRβ^-/-^) mice (B6.129P2^Tcrbtm1Mom^/J) that lack αβT cells. Briefly, whole spleens were resected, homogenized, and then subjected to density gradient centrifugation to isolate PBMC. Spleen cell cultures were initiated at a density of 5 × 10^6^ cells/ml in RPMI 1640 with 10% FBS, 2 mM/L L-glutamine, 100 U/ml penicillin, 100 U/ml streptomycin, and 50 μM 2-Mercaptoethanol. On the day of culture initiation (day 0), cells were transferred to tissue culture wells first coated with rat anti-mouse CD2 mAb clone RM2-5 (BD Biosciences). Recombinant mouse IFN-γ (1000 U/ml; R&D Systems) and recombinant mouse IL-12 (10 U/ml; R&D Systems) were then added. After 24 h (day 1), three volumes of fresh culture medium were added. Cultures were then stimulated with 10ng/ml anti-CD3 mAb clone 145-2C11 (BD Biosciences) and 300 U/ml mouse recombinant IL-2 (R&D Systems). Fresh medium with 10 U/ml human IL-2 (Roche Diagnostics) was added every 3 days. At day 8, cells were harvested. Purity of γδ T cells was assessed using a FACSCalibur flow cytometer (BD Biosciences) employing directly the conjugated hamster anti-mouse Abs CD3-allophycocyanin (clone 145-2C11), and TCRγδ-FITC (clone GL3; all obtained from BD Biosciences). Cell viability was determined by Propidium iodide uptake using flow cytometry.

### Flow cytometry

Immunophenotyping of mouse peripheral blood and spleen lymphocytes was performed on a BD LSRII flow cytometer (BD Biosciences; San Jose, CA). Mouse peripheral blood was labeled with fluorochrome-conjugated antibodies against CD3 and TCR-γδ, incubated in a red blood cell lysis preparation (FACS Lyse: BD Biosciences) and acquired in Tru-Count tubes (BD Biosciences). Spleen lymphocytes were obtained by mechanical disaggregation and purified by density-gradient centrifugation and were labeled with antibodies against CD3, TCRγδ, (R&D Systems; Minneapolis, MN), Vγ1.1, Vγ2, Vγ3, Vδ4, Vδ6.3 (Bio Legend; San Diego, CA), Vγ7, Vδ1 (from the laboratory of R.O.), and with Propidium iodide and Annexin V, (R&D Systems; Minneapolis, MN)

### Intracellular staining of cytokines

Peripheral blood leukocytes were obtained following RBC sedimentation in PBS with 2% dextran (weight/volume, MW 3000, Amersham) for 30 minutes at 37°C. Remaining red cells were lysed in an NH_4_Cl based isotonic buffer (FACSLyse, BD Biosciences; San Jose, CA), neutralized in cold PBS and incubated at 37°C for 4h at a concentration of 1 x 10^6^/mL in RPMI + Golgi Stop Protein Transport Inhibitor ± 20ng/ml of PMA and 1μg/ml of Ionomycin (Sigma Aldrich; St. Louis, MO). Cells were washed in cold PBS, incubated in PBS + 1μg of Fc Blocker for 15 minutes at 4°C and labeled with the following antibodies: anti-CD62L^FITC^, anti-CD3^PE^, anti- γδ TCR^BV421^, anti-CD27^BV510^ for 30 minutes at 4°C. After surface staining, cells were fixed and permeabilized in 250μM. Cytofix/Cytoperm (BD Biosciences) at 4°C, washed x 2 in BD Perm/Wash buffer and labeled with anti-cytokine antibodies for IL17^PerCP-Cy5.5^, IFNγ ^PECy7^, and IL4^APC^. Flow cytometry was performed on a FACS Canto analyzer (BD Biosciences) using FACS Diva software.

### Cytotoxicity assay

The GL261 target cells were plated (10,000 cells/well) into the wells of 96 well flat bottom plates over night to allow them to adhere. A separate aliquot of U251 cells were detached by incubating the cells in a versene buffer for 20 minutes. Detached and attached GL261 cells were labeled with CSFE (Molecular Probes/Invitrogen, Eugene, OR) directions. After 3 washes, the cells were incubated with the effector cells at 5:1, 10:1, 20:1, and 40:1 effector: target cell ratios in quadruplicate cultures. Triton X-100 (1%) treated cells served as the maximum release. GL261 cells incubated without any effector cells served as the spontaneous release values. After 6 hrs the supernatants was collected and analyzed on the Novostar Fluorometer/ Luminometer (BMG Labtech, Offenburg, Germany) for fluorescence.

Percent specific release was calculated as follows:

$$\%Specific\;Release\;=\;\frac{\;{Fluorescence}_{\left(experiment\right)}\;{-\;Fluorescence}_{\left(spontaneous\;release\right)}}{\;{Fluorescence}_{\left(maximum\right)}\;{-\;Fluorescence}_{\left(spontaneous\;release\right)}}\;\times \;100$$

Maximum release was determined by adding in 0.01% triton-X100.

### Cell lines and antibodies

The GL261 murine glioma cell line was obtained from NCI Division of Cancer Therapy Tumor Repository and stably transfected with firefly luciferase in the UAB Brain Tumor Tissue Facility. GL261 cells were maintained in a 1:1 mixture of Dulbecco's MEM and Ham's Nutrient Mixture F-12 containing 7% fetal calf serum and L-glutamine.

#### Statistical methods

Each series of mouse experiments was repeated three times with separate groups of mice and the data compiled for analysis. Likewise, a minimum of three repetitions was performed for each biologic assay. Descriptive statistics were used to express data from antigen expression, cell frequency, apoptosis, and cytotoxicity assays. Comparisons within groups were evaluated with non-parametric *t*-tests by Kruskal-Wallis and Kolmogorov-Smirnov analysis. Data from the cytotoxicity assays were analyzed using a student’s *t*-test. Values were considered significantly different at the *p*<0.05 levels. Animal survival included moribund animals that became unresponsive to mild external stimuli, which were euthanized and the date of death estimated to be the day the animal was killed. Kaplan-Meier analysis was used to estimate survival from tumor induction. The Mantel-Cox and Gehan-Breslow-Wilcoxon tests used to determine differences observed between treatment groups.

## Results

**Fig 1:**
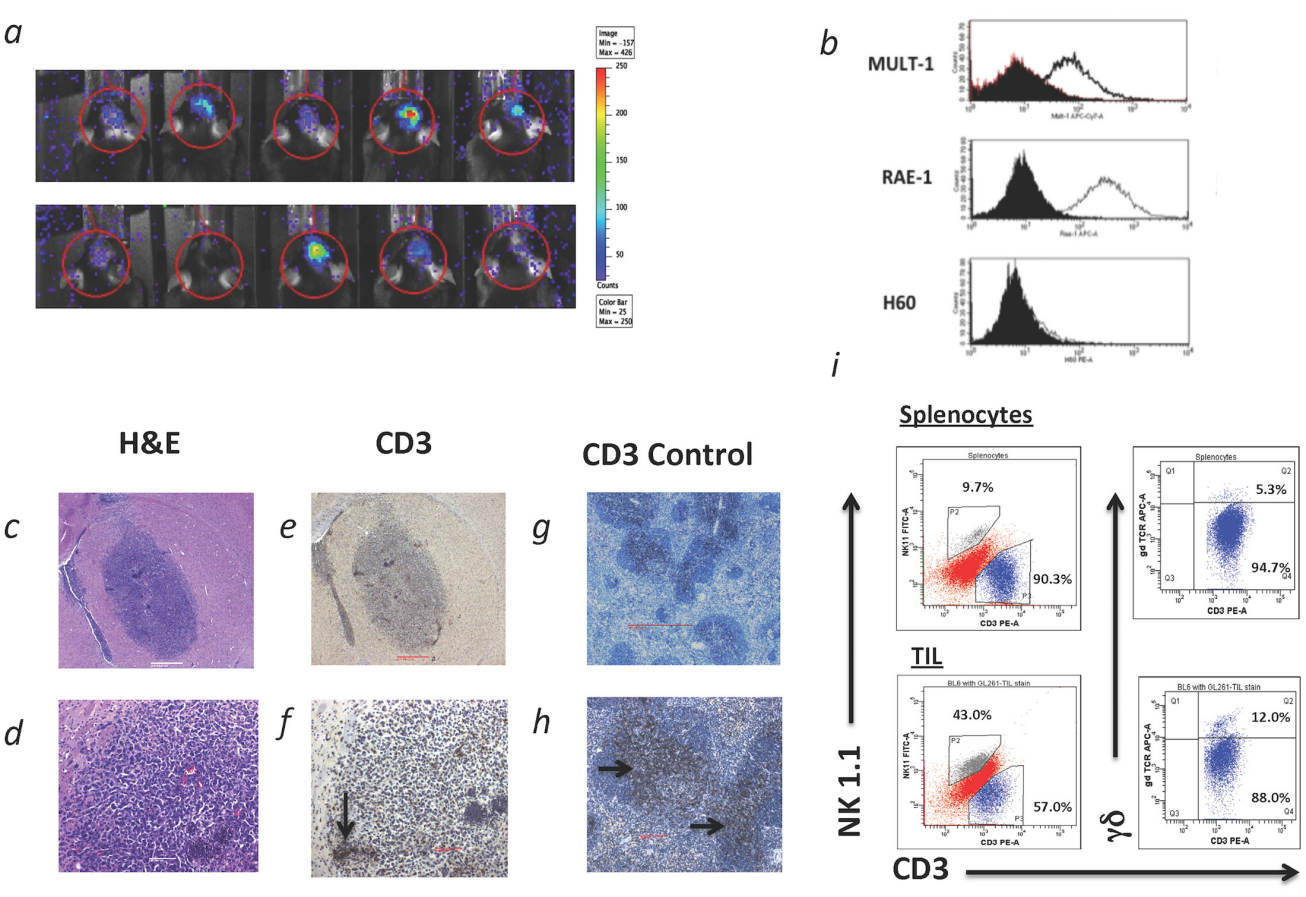
*(a)* Bioluminescence scans results obtained from ten representative mice five days following intracranial injection of GL261 glioma cells transduced with firefly luciferase. Note that tumor growth is detectable at 5 days post-injection. *(b)* Single parameter flow cytometric histograms from cultured GL261 cells after harvest, and labeling with the NKG2DL MULT-1, RAE-1, and H60. GL261 cells express the NKG2DLs MULT-1 and RAE-1, although H60 is not expressed due to a known genetic defect in this mouse strain. *(c-f)* Representative micrographs of a GL261-derived tumor from an athymic nude mouse killed at 10 days post-intracranial tumor injection (n = 20). Sections were fixed in formalin, embedded in paraffin, and sectioned for light microscopy. Low (40x)- and high-power (1000x) hematoxylin and eosin (H&E) staining *(c)* reveals the presence of small gliomas showing evidence of infiltration into the surrounding brain parenchyma and *(d)* characteristic features of malignant glioma with small necrotic centers and local infiltration. T cell infiltration assessed by immunohistochemical staining for CD3 *(e*, *f)* is sparse and scattered with localized clusters identified near microvasculature (arrow). *(g)* Low (20x) and *(h)* high-power (40x) shows WT C57BL/6 tonsil positive control for CD3 staining (arrows). *(i)* Comparison of splenocytes and tumor-infiltrating lymphocytes (TIL) from mice with 10 day old tumors (n = 5) following whole-body perfusion with saline. Note a higher proportion of NT to T cells and a higher proportion of γδ T cells of total CD3+ T cells in tumors relative to spleen.

Intracranial GL261 tumors grow rapidly in immunocompetent C56BL/6 WT mice and show characteristic features of high-grade gliomas. Tumors were detectable 5 days following intracranial injection (Fig 1a) and grew rapidly, resulting in a median survival of 30 days. Cultured GL261 cells expressed the γδ T cell target NKG2DLs MULT-1 and RAE-1 (Fig 1b). H60 is not expressed due to a known genetic defect in this mouse strain [29]. At 10–12 days following injection, neurologic symptoms had not yet manifested and tumors were small with characteristic features of malignant glioma such as pseudopalisading morphology, small necrotic centers, and local infiltration (Fig 1c and 1d). There was scattered evidence of T cell trafficking within the parenchyma of the tumor and localized clusters of T cells were occasionally observed surrounding the microvasculature (Fig 1e and 1f). Positive control staining for CD3 is shown in Fig 1g and 1h. Flow cytometric immunophenotyping of tumor-infiltrating lymphocytes (TIL) revealed an increased proportion of NK cells and γδ T cells in tumors when compared to splenocytes (Fig 1i), although absolute counts could not be determined in tissue.

**Fig 2:**
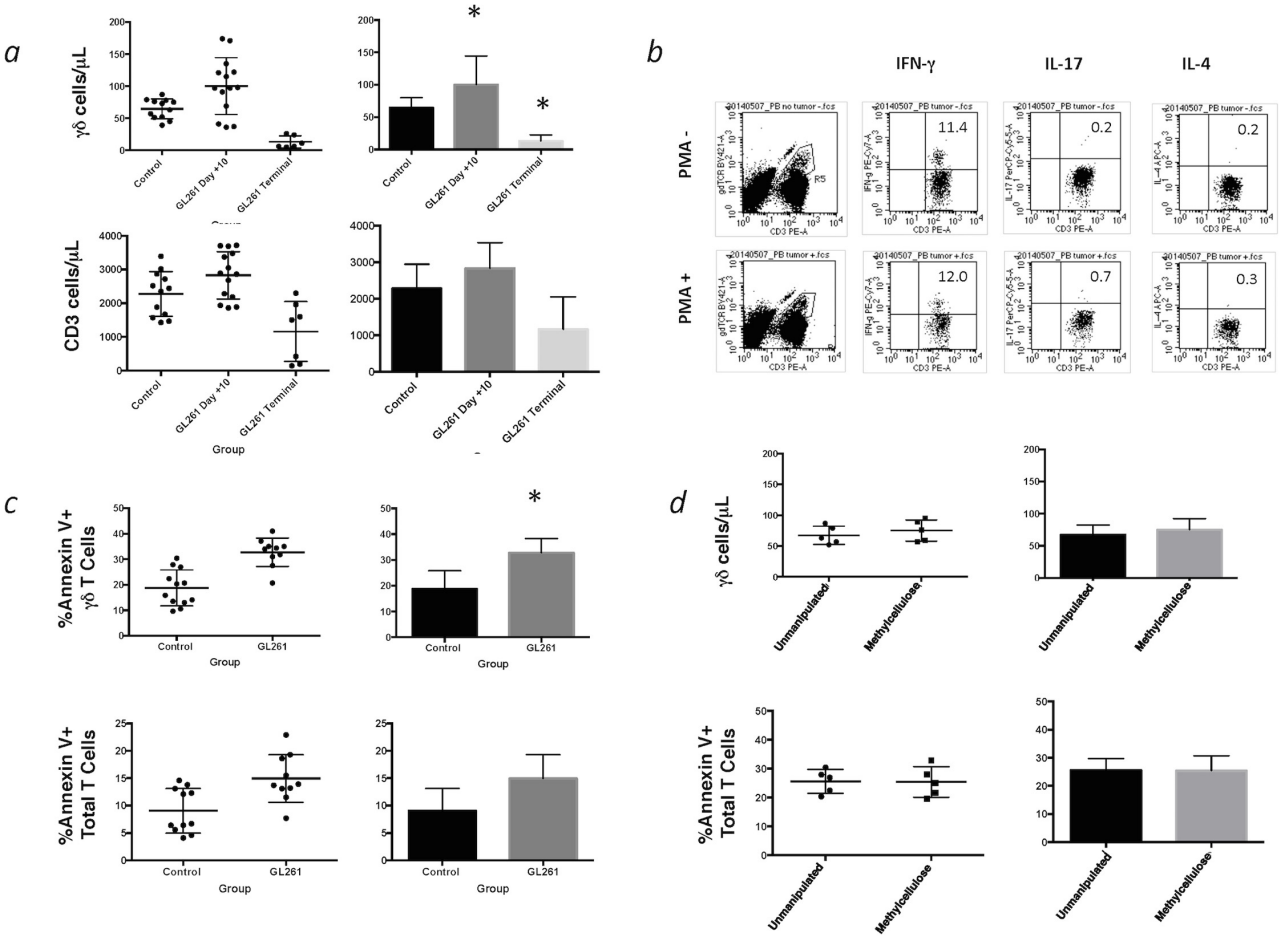
Circulating γδ T cell enumeration and function in tumor-bearing mice. *(a)* The circulating T cell count (distribution on left panels, x ± SD, right panels, * = p<0.05) was increased in tumor-bearing mice at 10 days post-GL261 injection independent of the total T cell count. Terminal γδ T cell counts in tumor-bearing mice also fell significantly lower than controls and day 10 glioma-bearing mice. *(b)* Approximately 12% of γδ T cells constitutively produced IFN-γ with no additional increase following PMA/Ionomycin stimulation. *(c)* Annexin V expression was upregulated on γδ T cells from glioma-bearing mice, also independently from the total T cell population indicating simultaneous γδ T cell proliferation and apoptosis likely due to activation-induced cell death (AICD). *(d)* The γδ T cell count and Annexin V expression were measured in unmanipulated (e.g. no intracranial injection) mice and 10 days following IC injection of the methylcellulose vehicle in which GL261 cells were suspended to determine if IC injection-related injury produced the same increase in γδ T cell count and Annexin V expression as tumor injection. There was no difference in either parameter between sham-injected mice and mice that received intracranial methylcellulose vehicle alone.

Circulating peripheral blood γδ T cells are increased during the early phase of tumor growth and depleted in the advanced stage. As shown in Fig 2a, the peripheral blood γδ T cell count for wild-type (WT) mice (n = 13) that did not receive tumor injections ranged from 39μL to 87/μL (64.58 ± 15.49). Most glioma-bearing mice (n = 17), at 10–12 days following tumor placement, showed a significant increase in peripheral blood γδ T cell counts over that of control mice (*p* = 0.003). The range was widely spread between 36/μL and 174/μL (100.40 ± 33.9) and did not result in a significant proportional increase over that observed for control mice (2.94% ± 1.24 in untreated mice vs. 3.06% ± 1.22 in tumor-bearing mice). Total CD3+ T cell count for control mice (2278 cells/μL± 662) was no different from glioma-bearing mice at 10–12 days post-tumor injection (2829 cells/μL ± 703; *p* = 0.424). Terminal γδ T cell counts (n = 12), obtained from tumor-bearing mice following development of significant neurologic symptoms, decreased by 78% from control and ranged from a low of 5/μL to 26/μL (13.00 ± 9.56) and were significantly lower than controls (p = 0.0001) and day 11±1 glioma-bearing mice (p<0.001). Total CD3+ T cell count for terminal mice also decreased by 51% from control T cell counts (1160 cells/μL ± 890) and were significantly lower than those from day 11±1 glioma-bearing mice (*p* = 0.0171), and approached a significant difference when compared to controls (*p* = 0.0648). The proportion of γδ T cells relative to the total CD3+ population (0.632% + 0.421) also fell significantly from that observed for controls (p<0.0001) and tumor-bearing mice (p<0.0001).

A small percentage of circulating γδ T cells from tumor-bearing mice were found to constitutively produce IFN-γ. The number of cells producing IFN-γ did not increase after a 4h stimulation with following PMA/Ionomycin. Only negligible production of IL-17 and IL-4 was observed (Fig 2b). A large proportion of circulating γδ T cells from tumor-bearing mice also expressed the apoptotic marker Annexin V at day 11 ± 1 (Fig 2c). Indeed, the percentage of γδ T cells expressing Annexin V was significantly increased in glioma-bearing mice (32.75 ± 5.86) over that of control mice (18.78 ± 7.04 p = 0.0019) while the total T cell population from glioma-bearing mice did not significantly differ from controls in this regard (14.94 + 4.34 vs. 9.06 + 4.07; p = 0.0886). Sham-injected mice that received intracranial methylcellulose vehicle alone also showed no difference in γδ T cell count or Annexin V expression at day 10 (Fig 2d).

**Fig 3:**
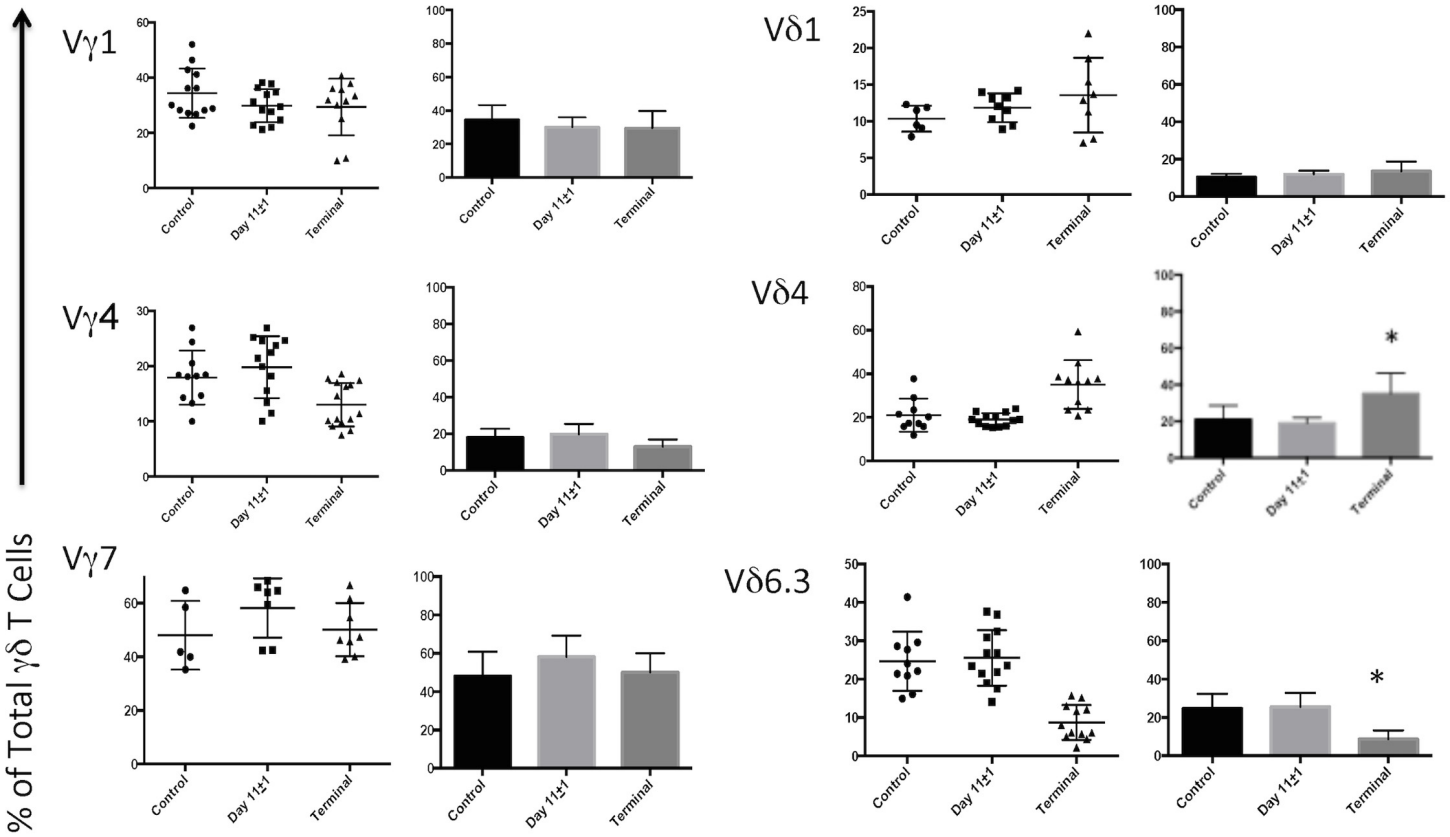
Flow cytometric evaluation of the γδ T cell receptor (TCR) repertoire in control vs. early and late stage tumor-bearing mice (distribution on left panels, x ± SD, right panels, * = p<0.05). There were no significant changes in the Vγ1, Vγ4, or Vγ7 subsets from controls either post-tumor injection day 11±1 or at end stage. Additionally, there were no changes in the proportions of Vδ1, Vδ4, and Vδ6.3 at day 10. A significant decrease of the %Vδ6.3 population at end stage was offset by a parallel increase in %Vδ4 cells. TCR subsets Vγ3 and Vδ3 were also measured, however, relative proportions were negligible *(data not shown)*.

We then examined the γδ TCR repertoire to assess whether the changes in circulating γδ T cell counts favored expansion and/or deletion of specific Vγ or Vδ TCR subset(s) (Fig 3). Significant changes in the Vγ1, Vγ4, or Vγ7 subsets from controls either post-tumor injection day 11±1 or at the termination of the experiment were not seen. Additionally, there were no changes in the proportions of Vδ1, Vδ4, and Vδ6.3 at day 11±1. We did notice a significant decrease of the Vδ6.3 population at end stage tumor growth vs. control (p = 0.0003) that was offset by a corresponding increase in the proportion of Vδ4 cells (p = 0.0410). The Vγ2Vδ4 IL-17 producing phenotype comprised only a small population of γδ T cells and did show a modest increase at post-tumor injection day 11±1 but did not reach statistical significance (p = 0.0954). TCR subset Vγ3 was also assessed, however, the relative proportion was negligible (data not shown).

**Fig 4:**
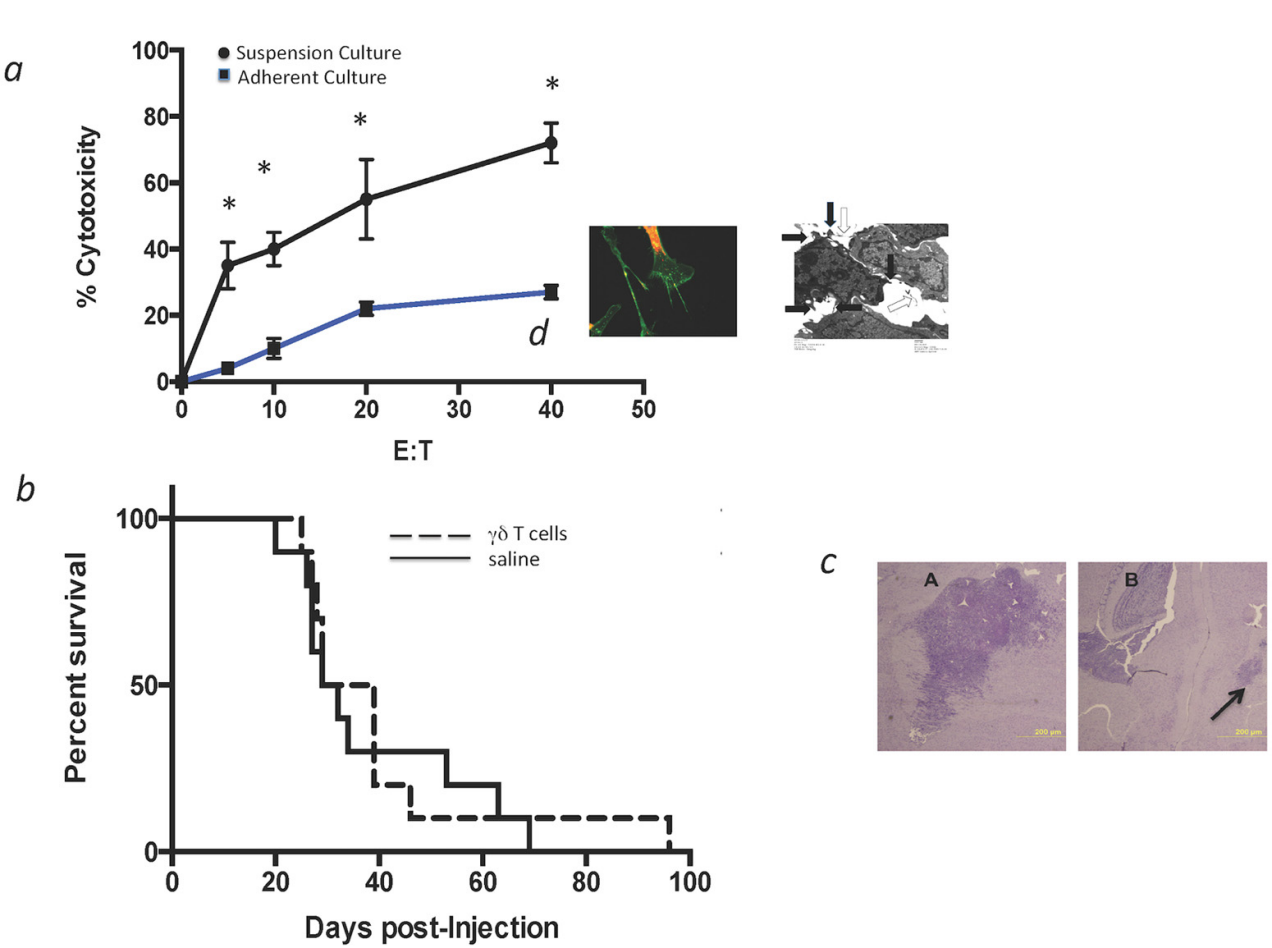
Cytotoxicity and growth inhibition assessment of activated γδ T cells against GL261 tumors. *(a)* Activated γδ T cells were incubated with GL261 cells either in suspension (closed circles) or attached to plastic (closed squares) show robust *in vitro* cytotoxicity against the non-adherent GL261 cells in a 4h standard assay (x ± SD, * = p<0.05). The insert is a high-power confocal micrograph of adherent GL261 cells cytoplasm stained with wheat germ agglutinin (green) showing microfilopodia spreading from the attached cells as well as tumor from an athymic nude mouse was harvested 10 days following GL261 cells, chopped and disaggregated in media, fixed/post-fixed in in glutaraldehyde and osmium tetroxide, dehydrated in ethanol, and embedded in epoxy resin. Filopodia (clear arrows) and microspikes (filled arrows) are seen on the cell surface in these transmission electron micrographs from tumor sections. *(b)* Injection of 5 x 10^5^ GL261 cells followed 15m later by 1.5 x 10^6^ activated γδ T cells (dashed line) did not improve median survival when compared to saline-injected mice (solid line). *(c)* Evidence of local tumor inhibition was noted in histologic examination. Representative histologic specimens show a much smaller tumor at the injection site (arrow) in the γδ T cells injected mouse (B) than from a control mouse (A) at 25 days.

*Ex vivo* activated γδ T cells are highly cytotoxic to GL261 glioma cells *in vitro* but have a variable effect on tumor progression or survival. *Ex vivo* cytolytic activity of γδ T cells was assessed against GL261 gliomas using standard methods. Cultured splenic γδ T cells were activated and expanded *ex vivo* from C57BL/6 TCRβ-deficient (TCRβ^-/-^) mice and co-cultured with suspended or attached GL261 cells as described above. Syngeneic γδ T cells were highly cytotoxic to GL261 cells in suspension as shown in Fig 4a. Adherent GL261 cells were more resistant to lysis, a finding previously reported by Hoa for human gliomas [30] and thought to be due to tumor cell membrane protrusions like microvilli/microspikes (Fig 4a insert) that create a physical barrier that prevents proper cytolytic processes to be fully delivered. *In vivo* cytotoxicity was then examined in a tumor growth inhibition assay. C57BL/6^WT^ mice were implanted with 5 x 10^5^ GL261 tumor cells in methylcellulose. Approximately 15 min following tumor placement, 1.5 x 10^6^ *ex vivo* activated syngeneic γδ T cells were stereotactically injected through the same burr hole at the same depth used for the GL261 cells injection. Control mice received 5 x 10^5^ GL261 tumor cells and were injected in the same manner with the saline vehicle. Mice were sacrificed at the onset of neurologic symptoms. All mice except one in the treatment group eventually succumbed to GL261 tumors. Median survival for mice that received the single treatment with *ex vivo* activated γδ T cells was improved by 9 days and generated one long-term survivor, but the therapy did not reach statistical significance (Fig 4b). Gross and histologic examination of brains from tumor-bearing mice that were injected with γδ T cells showed varying degrees of local tumor inhibition (Fig 4c) as compared to saline-treated control mice that uniformly developed large tumors at the injection site, suggesting that the comparative lack of *in vivo* cytotoxic effect and subsequent survival advantage may be a function of placement and migration of γδ T cells relative to the degree of tumor dispersal within the brain.

**Fig 5:**
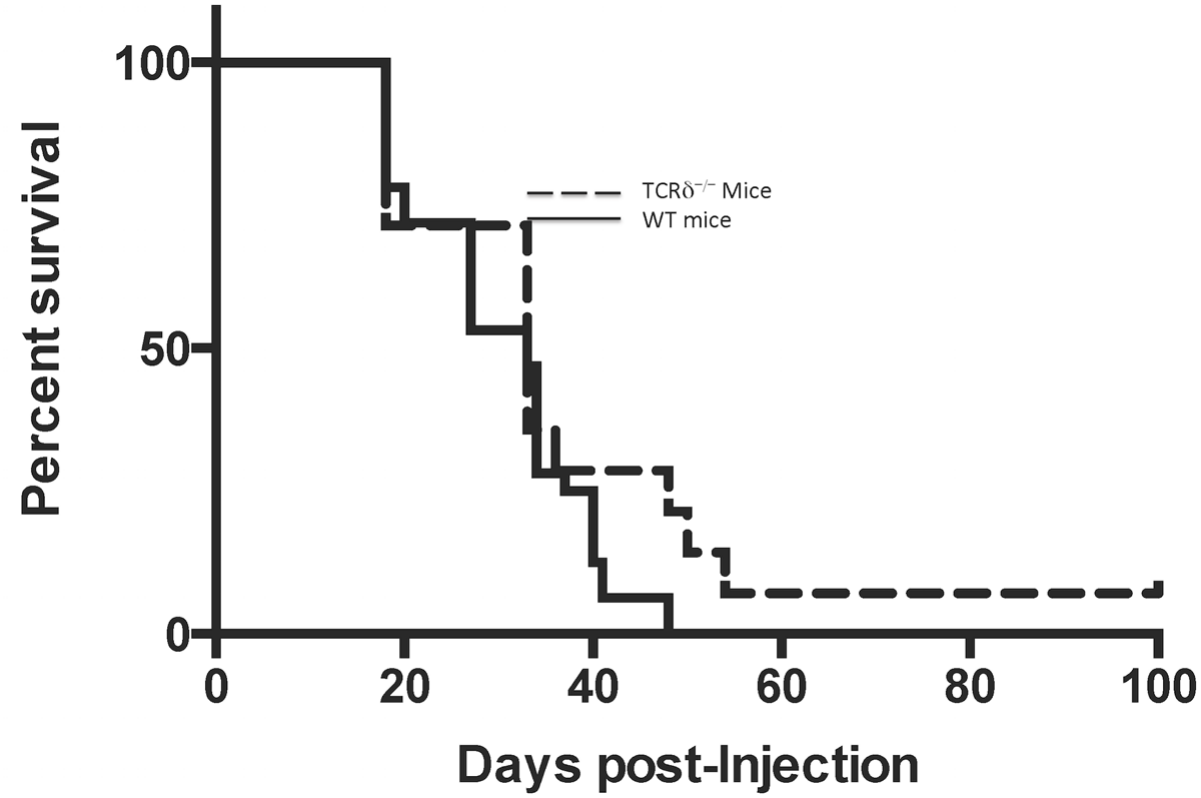
GL261-injected WT mice (solid line) and C57BL/6 TCR-δ^-/-^ mice (B6.129P2-TCR-δ^tm1Mom^/J) (dashed line) received intracranial injections of 5.0 x 10^5^ GL261 cells and were monitored for survival. Median survival for TCRδ^-/-^ mice was no different than WT, showing that the absence of γδ T cells does not significantly effect survival.

We then sought to determine whether γδ T cells contribute to slower tumor progression and improved survival, or alternatively exert a regulatory or immunosuppressive effect that accelerates tumor progression. C57BL/6^WT^ mice and C57BL/6 TCR-δ^-/-^ mice (B6.129P2-TCR-δ^tm1Mom^/J) that are genetically engineered to produce only αβ T cells received intracranial injections of 5 x 10^5^ GL261 glioma cells as described above (n = 10/group). Mice were sacrificed at the onset of neurologic symptoms, which was then considered the last day of survival. Gross and histopathologic examination of brains from both groups of mice revealed large infiltrative glioma tumors with substantial necrosis (data not shown). Overall survival did not achieve significance for either group (p = 0.156), suggesting a negligible γδ T cell effect on tumor progression (Fig 5).

## Discussion

In this report, we examine the dynamics of γδ T cell activity in a fully immunocompetent model of syngeneic high-grade glioma. Interestingly, this study revealed a generalized and transient increase of circulating γδ T cells in mice with small, aggressive intracranial GL261 gliomas and prior to the onset of neurologic symptoms. As a growing body of evidence suggests an important role for innate tumor recognition by γδ T cells [3,31–34], our findings raise the intriguing question of how circulating γδ T cells are activated and expanded early during the growth of high-grade gliomas. Although histologic examination of whole brains from tumor-bearing mice show scant evidence of tumor infiltration, we did document a proportional increase in innate TIL suggesting an early stage immune response. Local NK cell and marginally higher γδ T cell proportions within the 10-day tumors suggest infiltration and thereby a potential for of contact as a primary source of activation. The presence of activating soluble factor(s) could also stimulate γδ T cells, although we were unable to document any differences in proliferation of γδ T cells in a culture of C57BL/6 mouse spleen cells in GL261-conditioned medium versus spleen cells cultured in unmodified commercial medium (data not shown). Indeed, circulating cytokines in GL261 tumor-bearing mice are more likely to be immunosuppressive [35].

The TCR repertoire for γδ T cells collected at day 11±1 was no different than that found in control mice, suggesting that the γδ T cell expansion observed in the early period of tumor growth was nonspecific in nature. IFN-γ producing γδ T cells formed approximately 12% of the total population and did not increase with PMA stimulation. IFN-γ has generally been associated with both Vγ1+ and Vγ4+ cells, which have been shown to be protective against some solid malignancies [36,37] Interestingly, the absolute Vδ4+ T cell count in mice with an overwhelming tumor burden was significantly increased over that from control mice, an observation that will require further study. Vδ4+ cells may resist AICD and persist following stimulation similar to the human Vδ1+ persistence over the more sensitive Vγ9Vδ2 population[38,39].

We also documented a population of Vγ1+ γδ T cells, which have been associated with a suppressive IL-4 population. Interestingly, we did not observe substantial IL-4 production in quiescent or stimulated γδ T cells. Additionally, GL261-derived tumors are known to produce TGFβ, raising the expectation that IL-17 producing γδ T cells responsive to TGFβ may be more evident [40]. The lack of a significant percentage of IL-4 and IL-17 producing γδ T cells and the absence of a survival advantage in tumor-bearing TCRδ^-/-^ mice appears to indicate that γδ T cells in this model do not have a significant immunoregulatory role. More importantly, the significant increase in Annexin-V observed in proliferating γδ T cells indicates that many cells were in a pre-apoptotic state likely with deteriorating function.

Functional T cell exhaustion has been recently reviewed [41] and is characterized by lack of robust proliferative response, loss of cytotoxic activity, and other critical functions. It appears likely that the depletion and functional anergy of γδ T cells observed in this study and in patients is due to T cell exhaustion progressing to activation induced cell death (AICD). One important difference is the contrast between the Vδ4 and Vδ6.3 populations at end stage detailed Fig 3. Our laboratory (RO) in particular has studied a Vγ4Vδ 4+ subset having nearly invariant TCR junctions that shows a strong bias to secrete IL-17[42]. As for Vδ6.3+ cells, an NKT-like subset of γδ T cells bearing a nearly invariant Vγ1Vδ6.3 TCR has been described that, like αβ iNKT cells, can produce both IL-4 and IFNγ[43]. So we could speculate that these differences might reflect a failed attempt by the mice to control the glioma by increasing tumor-suppressive Vγ4Vδ4+ cells that can be stimulated to produce IL-17, and decreasing tumor-promoting Vγ1Vδ6.3+ cells that can be stimulated to produce IL-4. An attempt to correlate these findings in co-culture studies revealed only that repetitive stimulation of syngeneic γδ T cells with tumor failed to establish a specific clone resistant to activation induced cell death.

In similar studies, we showed that γδ T cells readily infiltrate 4T1-derived breast tumors in mice [44], and that the 4T1 cell line drives γδ T cells to apoptosis *in vitro*, a process that is contact-dependent. Additionally, GL261-derived TGF-β1 expression and secretion is increased following exposure to conditioned media derived from activated T cells, suggesting that this immunosuppressive cytokine is not constitutively expressed by GL261 but is upregulated in response to immune activation[35]. This finding suggests the intriguing concept that tumor-activated γδ T cells may provoke the release of immunosuppressive cytokines that ultimately result in their demise.

Previous studies in our laboratory and others have also shown that γδ T cells are generally depleted in cancer patients including patients with high-grade gliomas [23,39,45], although the dynamics of this depletion has not previously been described. These observations, together with data showing a progressive loss of γδ T cells and function in mice and humans with GBM and other cancers, appear to indicate that the depletion of γδ T cells may be the end result of a failed anti-tumor response resulting in their clearance from the circulation by AICD as suggested by Ferrarini [46], who first showed that γδ T cells are activated in culture upon contact with Daudi lymphoma cells but subsequently succumb to AICD.

Despite the expression of activating NKG2D ligands on 10-day tumors, we observed only sparse evidence of T lymphocyte invasion into the tumor parenchyma. Indeed, transgenic mice devoid of γδ T cells fared no better or worse than fully immunocompetent mice after intracranial challenge with GL261 cell line tumors. These findings suggest that an immunosuppressive microenvironment prevents substantial T cell infiltration and therefore protects the tumor from a cytotoxic response. With this in mind, we then sought to determine if γδ T cells could inhibit tumor progression if placed in a minimal disease environment. *Ex vivo*-activated γδ T cells obtained from syngeneic C57BL/6 mice bearing the *Tcrβ* ^*tm1Mom*^ mutation exhibited significant cytotoxicity to GL261 glioma cells in a standardized *in vitro* 4h suspension assay. Direct injection of cultured syngeneic γδ T cells into the tumor bed extended median survival by nine days over that of control mice and generated one long-term survivor. A variable degree of local inhibition of tumor growth was also evident in mice that received intracranial γδ T cells. Survival did not reach statistical significance, however, and appeared to be dependent on the degree of dispersal of tumor at the time of injection. Examination of histologic sections suggested that variable suppression of local tumor growth was confined to the area of the brain in which γδ T cells were injected, but was insufficient to maintain a durable effect on overall tumor progression.

Immunosuppression can be multifactorial and promote tumor escape at any stage of growth. Microfilopodia (Fig 3a) Glioma cell lines express complex cell surface projections such as microvilli, filopodia and microspikes. These projections are displayed on glioma cells and inhibit human CTLs, LAK cells, CAR-T cells and γδ T cells and can dampen anti-tumor efficacy by physical inhibition of cytotoxic T cell binding [30] and subsequent cytotoxicity regardless of tumor size. In this study, saw identical effects with the mouse GL261 cells with γδ T cells as shown in (Figs 3a and 4a). Microvilli and filopodia are internally supported by fascin-1. When knocked down fascin-1 by shRNA, U251 cells lose their membrane projections, and are killed more effectively by CTL[47–49].

Glioma-derived matrix metalloproteinase and TGFβ also suppress tumor cell NKG2DL expression *in vivo* [50] and facilitate immune escape. Immunosuppressive cytokines, inhibitory factors, monocyte-derived suppressor cells, and other factors such as checkpoint inhibitory membrane proteins limit the immune response via multiple mechanisms that impede T cell activation and migration [51–53], all forming a substantial defense against any immune response generated by the host.

Taken together, our findings show a dynamic of early γδ T cell proliferation and apoptosis followed by profound γδ T cell depletion in mice implanted with syngeneic GL261 high-grade gliomas. The biologic mechanism by which this γδ T cell expansion occurs remains a subject for further investigation that ultimately could lead to an understanding of γδ T cell-based tumor response and strategies that augment and sustain the immune response to high-grade gliomas.

## References

[1] Brenner MB, McLean J, Dialynas DP, Strominger JL, Smith JA, Owen FL, et al (1986) Identification of a putative second T-cell receptor. Nature 322: 145–149. 3755221 10.1038/322145a0

[2] Shin S, El-Diwany R, Schaffert S, Adams EJ, Garcia KC, Pereira P, et al (2005) Antigen recognition determinants of gammadelta T cell receptors. Science 308: 252–255. 15821090 10.1126/science.1106480

[3] Girardi M, Oppenheim DE, Steele CR, Lewis JM, Glusac E, Filler R, et al (2001) Regulation of cutaneous malignancy by gammadelta T cells. Science 294: 605–609. 11567106 10.1126/science.1063916

[4] Liu Z, Eltoum IE, Guo B, Beck BH, Cloud GA, Lopez RD (2008) Protective immunosurveillance and therapeutic antitumor activity of gammadelta T cells demonstrated in a mouse model of prostate cancer. J Immunol 180: 6044–6053. 18424725 10.4049/jimmunol.180.9.6044

[5] Ferrarini M, Pupa SM, Zocchi MR, Rugarli C, Menard S (1994) Distinct pattern of HSP72 and monomeric laminin receptor expression in human lung cancers infiltrated by gamma/delta T lymphocytes. Int J Cancer 57: 486–490. 7514151 10.1002/ijc.2910570408

[6] Choudhary A, Davodeau F, Moreau A, Peyrat MA, Bonneville M, Jotereau F (1995) Selective lysis of autologous tumor cells by recurrent gamma delta tumor-infiltrating lymphocytes from renal carcinoma. J Immunol 154: 3932–3940. 7706731

[7] Zhao X, Wei YQ, Kariya Y, Teshigawara K, Uchida A (1995) Accumulation of gamma/delta T cells in human dysgerminoma and seminoma: roles in autologous tumor killing and granuloma formation. Immunol Invest 24: 607–618. 7622197 10.3109/08820139509066861

[8] Bagot M, Heslan M, Dubertret L, Roujeau JC, Tourine R, Levy JP (1985) Antigen-presenting properties of human epidermal cells compared with peripheral blood mononuclear cells. British Journal of Dermatology 113 (suppl. 28): 55 2410006 10.1111/j.1365-2133.1985.tb15626.x

[9] Fisch P, Malkovsky M, Kovats S, Sturm E, Braakman E, Klein BS, et al (1990) Recognition by human V gamma 9/V delta 2 T cells of a GroEL homolog on Daudi Burkitt's lymphoma cells. Science 250: 1269–1273. 1978758 10.1126/science.1978758

[10] Bukowski JF, Morita CT, Tanaka Y, Bloom BR, Brenner MB, Band H (1995) V gamma 2V delta 2 TCR-dependent recognition of non-peptide antigens and Daudi cells analyzed by TCR gene transfer. Journal of Immunology 154: 998–1006. 7529807

[11] Yamaguchi T, Fujimiya Y, Suzuki Y, Katakura R, Ebina T (1997) A simple method for the propagation and purification of gamma delta T cells from the peripheral blood of glioblastoma patients using solid-phase anti-CD3 antibody and soluble IL-2. J Immunol Methods 205: 19–28. 9236911 10.1016/s0022-1759(97)00062-8

[12] Yamaguchi T, Suzuki Y, Katakura R, Ebina T, Yokoyama J, Fujimiya Y (1998) Interleukin-15 effectively potentiates the in vitro tumor-specific activity and proliferation of peripheral blood gammadeltaT cells isolated from glioblastoma patients. Cancer Immunol Immunother 47: 97–103. 9769118 10.1007/s002620050509 PMC11037329

[13] Schilbach KE, Geiselhart A, Wessels JT, Niethammer D, Handgretinger R (2000) Human gammadelta T lymphocytes exert natural and IL-2-induced cytotoxicity to neuroblastoma cells. J Immunother 23: 536–548. 11001547 10.1097/00002371-200009000-00004

[14] Ferrarini M, Ferrero E, Dagna L, Poggi A, Zocchi MR (2002) Human gammadelta T cells: a nonredundant system in the immune-surveillance against cancer. Trends Immunol 23: 14–18. 11801449 10.1016/s1471-4906(01)02110-x

[15] Leca G, Vita N, Maiza H, Fasseu M, Bensussan A (1994) A monoclonal antibody to the Hodgkin's disease-associated antigen CD30 induces activation and long-term growth of human autoreactive gamma delta T cell clone. Cell Immunol 156: 230–239. 8200037 10.1006/cimm.1994.1167

[16] Godder KT, Henslee-Downey PJ, Mehta J, Park BS, Chiang KY, Abhyankar S, et al (2007) Long term disease-free survival in acute leukemia patients recovering with increased gammadelta T cells after partially mismatched related donor bone marrow transplantation. Bone Marrow Transplant 39: 751–757. 17450185 10.1038/sj.bmt.1705650

[17] Lamb LS Jr., Henslee-Downey PJ, Parrish RS, Godder K, Thompson J, Lee C, et al (1996) Increased frequency of TCR gamma delta + T cells in disease-free survivors following T cell-depleted, partially mismatched, related donor bone marrow transplantation for leukemia. Journal of Hematotherapy 5: 503–509. 8938522 10.1089/scd.1.1996.5.503

[18] Lamb LS Jr., Gee AP, Hazlett LJ, Musk P, Parrish RS, O'Hanlon TP, et al (1999) Influence of T cell depletion method on circulating gammadelta T cell reconstitution and potential role in the graft-versus-leukemia effect. Cytotherapy 1: 7–19. 19746645 10.1080/0032472031000141295

[19] Bauer S, Groh V, Wu J, Steinle A, Phillips JH, Lanier LL, et al (1999) Activation of NK cells and T cells by NKG2D, a receptor for stress-inducible MICA [see comments]. Science 285: 727–729. 10426993 10.1126/science.285.5428.727

[20] Friese MA, Platten M, Lutz SZ, Naumann U, Aulwurm S, Bischof F, et al (2003) MICA/NKG2D-mediated immunogene therapy of experimental gliomas. Cancer Res 63: 8996–9006. 14695218

[21] Wu A, Wiesner S, Xiao J, Ericson K, Chen W, Hall WA, et al (2007) Expression of MHC I and NK ligands on human CD133+ glioma cells: possible targets of immunotherapy. J Neurooncol 83: 121–131. 17077937 10.1007/s11060-006-9265-3

[22] Bryant NL, Gillespie GY, Lopez RD, Markert JM, Cloud GA, Langford CP, et al (2011) Preclinical evaluation of ex vivo expanded/activated gammadelta T cells for immunotherapy of glioblastoma multiforme. Journal of neuro-oncology 101: 179–188. 10.1007/s11060-010-0245-2 20532954 PMC13015848

[23] Bryant NL, Suarez-Cuervo C, Gillespie GY, Markert JM, Nabors LB, Meleth S, et al (2009) Characterization and immunotherapeutic potential of gammadelta T-cells in patients with glioblastoma. Neuro Oncol 11: 357–367. 10.1215/15228517-2008-111 19211933 PMC2743216

[24] Fujimiya Y, Suzuki Y, Katakura R, Miyagi T, Yamaguchi T, Yoshimoto T, et al (1997) In vitro interleukin 12 activation of peripheral blood CD3(+)CD56(+) and CD3(+)CD56(-) gammadelta T cells from glioblastoma patients. Clin Cancer Res 3: 633–643. 9815731

[25] Bryant NA, Rash AS, Woodward AL, Medcalf E, Helwegen M, Wohlfender F, et al (2011) Isolation and characterisation of equine influenza viruses (H3N8) from Europe and North America from 2008 to 2009. Vet Microbiol 147: 19–27. 10.1016/j.vetmic.2010.05.040 20580170

[26] Hayday A, Tigelaar R (2003) Immunoregulation in the tissues by gammadelta T cells. Nat Rev Immunol 3: 233–242. 12658271 10.1038/nri1030

[27] Pennington DJ, Vermijlen D, Wise EL, Clarke SL, Tigelaar RE, Hayday AC (2005) The integration of conventional and unconventional T cells that characterizes cell-mediated responses. Adv Immunol 87: 27–59. 16102571 10.1016/S0065-2776(05)87002-6

[28] Peng G, Wang HY, Peng W, Kiniwa Y, Seo KH, Wang RF (2007) Tumor-infiltrating gammadelta T cells suppress T and dendritic cell function via mechanisms controlled by a unique toll-like receptor signaling pathway. Immunity 27: 334–348. 17656116 10.1016/j.immuni.2007.05.020

[29] Malarkannan S, Shih PP, Eden PA, Horng T, Zuberi AR, Christianson G, et al (1998) The molecular and functional characterization of a dominant minor H antigen, H60. J Immunol 161: 3501–3509. 9759870

[30] Hoa N, Ge L, Kuznetsov Y, McPherson A, Cornforth AN, Pham JT, et al (2010) Glioma Cells Display Complex Cell Surface Topographies That Resist the Actions of Cytolytic Effector Lymphocytes. J Immunol. 10.4049/jimmunol.1001526 20855883

[31] Kabelitz D, Wesch D, He W (2007) Perspectives of gammadelta T cells in tumor immunology. Cancer Res 67: 5–8. 17210676 10.1158/0008-5472.CAN-06-3069

[32] Lamb LS Jr., Lopez RD (2005) gammadelta T cells: a new frontier for immunotherapy? Biology of blood and marrow transplantation: journal of the American Society for Blood and Marrow Transplantation 11: 161–168. 15744234 10.1016/j.bbmt.2004.11.015

[33] Dieli F, Vermijlen D, Fulfaro F, Caccamo N, Meraviglia S, Cicero G, et al (2007) Targeting human {gamma}delta} T cells with zoledronate and interleukin-2 for immunotherapy of hormone-refractory prostate cancer. Cancer Res 67: 7450–7457. 17671215 10.1158/0008-5472.CAN-07-0199 PMC3915341

[34] Wrobel P, Shojaei H, Schittek B, Gieseler F, Wollenberg B, Kalthoff H, et al (2007) Lysis of a broad range of epithelial tumour cells by human gamma delta T cells: involvement of NKG2D ligands and T-cell receptor- versus NKG2D-dependent recognition. Scand J Immunol 66: 320–328. 17635809 10.1111/j.1365-3083.2007.01963.x

[35] Ksendzovsky A, Feinstein D, Zengou R, Sharp A, Polak P, Lichtor T, et al (2009) Investigation of immunosuppressive mechanisms in a mouse glioma model. Journal of neuro-oncology 93: 107–114. 10.1007/s11060-009-9884-6 19430886

[36] Gao Y, Yang W, Pan M, Scully E, Girardi M, Augenlicht LH, et al (2003) Gamma delta T cells provide an early source of interferon gamma in tumor immunity. J Exp Med 198: 433–442. 12900519 10.1084/jem.20030584 PMC2194096

[37] He W, Hao J, Dong S, Gao Y, Tao J, Chi H, et al (2010) Naturally activated V gamma 4 gamma delta T cells play a protective role in tumor immunity through expression of eomesodermin. J Immunol 185: 126–133. 10.4049/jimmunol.0903767 20525896 PMC3813958

[38] Schilbach K, Frommer K, Meier S, Handgretinger R, Eyrich M (2008) Immune response of human propagated gammadelta-T-cells to neuroblastoma recommend the Vdelta1+ subset for gammadelta-T-cell-based immunotherapy. J Immunother 31: 896–905. 10.1097/CJI.0b013e31818955ad 18832998

[39] Meeh PF, King M, O'Brien RL, Muga S, Buckhalts P, Neuberg R, et al (2006) Characterization of the gammadelta T cell response to acute leukemia. Cancer Immunol Immunother 55: 1072–1080. 16328383 10.1007/s00262-005-0094-6 PMC11031079

[40] Do JS, Visperas A, O'Brien RL, Min B (2012) CD4 T cells play important roles in maintaining IL-17-producing gammadelta T-cell subsets in naive animals. Immunol Cell Biol 90: 396–403. 10.1038/icb.2011.50 21647171 PMC3170686

[41] Akbar AN, Henson SM (2011) Are senescence and exhaustion intertwined or unrelated processes that compromise immunity? Nature reviews Immunology 11: 289–295. 10.1038/nri2959 21436838

[42] Roark CL, Huang Y, Jin N, Aydintug MK, Casper T, Sun D, et al (2013) A canonical Vgamma4Vdelta4+ gammadelta T cell population with distinct stimulation requirements which promotes the Th17 response. Immunol Res 55: 217–230. 10.1007/s12026-012-8364-9 22961659 PMC3543513

[43] Hao J, Dong S, Xia S, He W, Jia H, Zhang S, et al (2011) Regulatory role of Vgamma1 gammadelta T cells in tumor immunity through IL-4 production. J Immunol 187: 4979–4986. 10.4049/jimmunol.1101389 21987661

[44] Beck BH, Kim HG, Kim H, Samuel S, Liu Z, Shrestha R, et al (2010) Adoptively transferred ex vivo expanded gammadelta-T cells mediate i*n vivo* antitumor activity in preclinical mouse models of breast cancer. Breast cancer research and treatment 122: 135–144. 10.1007/s10549-009-0527-6 19763820 PMC2883655

[45] Argentati K, Re F, Serresi S, Tucci MG, Bartozzi B, Bernardini G, et al (2003) Reduced number and impaired function of circulating gamma delta T cells in patients with cutaneous primary melanoma. Journal of Investigative Dermatology 120: 829–834. 12713589 10.1046/j.1523-1747.2003.12141.x

[46] Ferrarini M, Heltai S, Toninelli E, Sabbadini MG, Pellicciari C, Manfredi AA (1995) Daudi lymphoma killing triggers the programmed death of cytotoxic V gamma 9/V delta 2 T lymphocytes. Journal of Immunology 154: 3704–3712. 7706713

[47] Hwang JH, Smith CA, Salhia B, Rutka JT (2008) The role of fascin in the migration and invasiveness of malignant glioma cells. Neoplasia 10: 149–159. 18283337 10.1593/neo.07909 PMC2244690

[48] Machesky LM, Li A (2010) Fascin: Invasive filopodia promoting metastasis. Commun Integr Biol 3: 263–270. 20714410 10.4161/cib.3.3.11556 PMC2918773

[49] Li A, Dawson JC, Forero-Vargas M, Spence HJ, Yu X, Konig I, et al (2010) The actin-bundling protein fascin stabilizes actin in invadopodia and potentiates protrusive invasion. Curr Biol 20: 339–345. 10.1016/j.cub.2009.12.035 20137952 PMC3163294

[50] Eisele G, Wischhusen J, Mittelbronn M, Meyermann R, Waldhauer I, Steinle A, et al (2006) TGF-beta and metalloproteinases differentially suppress NKG2D ligand surface expression on malignant glioma cells. Brain 129: 2416–2425. 16891318 10.1093/brain/awl205

[51] Smyth MJ, Strobl SL, Young HA, Ortaldo JR, Ochoa AC (1991) Regulation of lymphokine-activated killer activity and pore-forming protein gene expression in human peripheral blood CD8+ T lymphocytes. Inhibition by transforming growth factor-beta. J Immunol 146: 3289–3297. 1827481

[52] Inge TH, McCoy KM, Susskind BM, Barrett SK, Zhao G, Bear HD (1992) Immunomodulatory effects of transforming growth factor-beta on T lymphocytes. Induction of CD8 expression in the CTLL-2 cell line and in normal thymocytes. J Immunol 148: 3847–3856. 1602133

[53] Jachimczak P, Bogdahn U, Schneider J, Behl C, Meixensberger J, Apfel R, et al (1993) The effect of transforming growth factor-beta 2-specific phosphorothioate-anti-sense oligodeoxynucleotides in reversing cellular immunosuppression in malignant glioma. J Neurosurg 78: 944–951. 8487077 10.3171/jns.1993.78.6.0944

